# Mesenchymal stromal cell‐derived syndecan‐2 regulates the immune response during sepsis to foster bacterial clearance and resolution of inflammation

**DOI:** 10.1111/febs.16154

**Published:** 2021-08-15

**Authors:** Junwen Han, Yuanyuan Shi, Gareth Willis, Jewel Imani, Min‐Young Kwon, Gu Li, Ehab Ayaub, Sailaja Ghanta, Julie Ng, Narae Hwang, Konstantin Tsoyi, Souheil El‐Chemaly, Stella Kourembanas, S. Alex Mitsialis, Ivan O. Rosas, Xiaoli Liu, Mark A. Perrella

**Affiliations:** ^1^ Division of Pulmonary and Critical Care Medicine Department of Medicine Brigham and Women's Hospital and Harvard Medical School Boston MA USA; ^2^ School of Life Sciences Beijing University of Chinese Medicine China; ^3^ Division of Newborn Medicine Department of Pediatrics Boston Children's Hospital and Harvard Medical School MA USA; ^4^ Department of Pediatric Newborn Medicine Brigham and Women's Hospital and Harvard Medical School Boston MA USA; ^5^ Division of Pulmonary, Critical Care and Sleep Medicine Department of Medicine Baylor College of Medicine Houston TX USA

**Keywords:** extracellular vesicles, macrophage polarization, mesenchymal stromal cells, resolution of inflammation, sepsis

## Abstract

Sepsis is a life‐threatening process related to a dysregulated host response to an underlying infection, which results in organ dysfunction and poor outcomes. Therapeutic strategies using mesenchymal stromal cells (MSCs) are under investigation for sepsis, with efforts to improve cellular utility. Syndecan (SDC) proteins are transmembrane proteoglycans involved with cellular signaling events including tissue repair and modulating inflammation. Bone marrow‐derived human MSCs express syndecan‐2 (SDC2) at a level higher than other SDC family members; thus, we explored SDC2 in MSC function. Administration of human MSCs silenced for SDC2 in experimental sepsis resulted in decreased bacterial clearance, and increased tissue injury and mortality compared with wild‐type MSCs. These findings were associated with a loss of resolution of inflammation in the peritoneal cavity, and higher levels of proinflammatory mediators in organs. MSCs silenced for SDC2 had a decreased ability to promote phagocytosis of apoptotic neutrophils by macrophages in the peritoneum, and also a diminished capability to convert macrophages from a proinflammatory to a proresolution phenotype via cellular or paracrine actions. Extracellular vesicles are a paracrine effector of MSCs that may contribute to resolution of inflammation, and their production was dramatically reduced in SDC2‐silenced human MSCs. Collectively, these data demonstrate the importance of SDC2 for cellular and paracrine function of human MSCs during sepsis.

## Graphical abstract

Silencing of syndecan‐2 (shSDC2) in human mesenchymal stromal cells (hMSCs) leads to less effective bacterial clearance, more tissue injury due to persistent inflammation, and increased mortality in experimental sepsis. shSDC2 hMSCs have reduced efferocytosis and altered macrophage M2 polarization, which is associated with decreased production of extracellular vesicles.

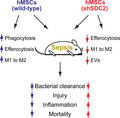

**Abbreviations**
AA: arachidonic acid
CLP: cecal ligation and puncture
CM: conditioned medium
DHA: docosahexaenoic acid
EVs: extracellular vesicles
hMSCs: human mesenchymal stromal cells
IFN‐γ: interferon gamma
IL‐6: interleukin‐6
LOX: lipoxygenase
LPS: lipopolysaccharide
MCP‐1: monocyte chemoattractant protein‐1
MSCs: mesenchymal stromal cells
NTA: nanoparticle tracking analysis
qRT‐PCR: quantitative real‐time PCR
SDC: syndecan
SDC2: syndecan‐2
shSCR: scrambled control short hairpin construct
shSDC2: silenced SDC2 short hairpin construct
SPMs: specialized proresolving lipid mediators
TEM: transmission electron microscopy
TNF‐α: tumor necrosis factor alpha
TUNEL: terminal deoxynucleotide transferase‐mediated dUTP nick end‐labeling

## Introduction

Sepsis is defined as life‐threatening organ dysfunction caused by a dysregulated host response to infection [1]. While comorbidities of the host, genetic determinants, and environmental or other factors contribute to this dysregulated response resulting in sepsis, the ability of the immune system to clear the inciting pathogen is critical. Without control of the underlying infection, and the appropriate resolution of the inflammatory response, collateral organ injury occurs contributing to organ dysfunction and a poor outcome. The interaction of innate immune cells, including neutrophils and macrophages, allows the host to efficiently clear pathogens and to return to homeostasis [2, 3].

Due to the challenges of therapy for the sepsis syndrome, and the fact that management remains predominantly supportive, new advances are being explored including the use of cell‐based therapies [4, 5, 6, 7, 8]. Mesenchymal stromal cells (MSCs) [9] have shown promise in experimental models of sepsis [8]. The immune evasive properties of MSCs allow the use of allogeneic cells in humans and also permit the use of human cells in mouse models of disease for preclinical investigation [10]. A critical property of MSCs, or their paracrine components [11], is modulation of the immune response that allows clearance of the invading organism(s) and limits tissue injury during sepsis [12]. While our laboratory and others have demonstrated that MSCs improve outcomes in experimental models of sepsis in mice [13, 14, 15, 16, 17, 18, 19], we seek to further understand how MSCs control the immune response during sepsis, with a special interest in the interaction between macrophages and neutrophils.

In an effort to identify a more homogeneous population of human MSCs, which does not require plastic adherence in culture and *in vitro* cell surface marker profiles and differentiation assays, investigators have recently evaluated the use of syndecan‐2 (SDC2 or CD362) as a cell surface marker of a subpopulation of MSCs. SDC2^+^ bone marrow‐derived human MSCs were shown to decrease the severity of *Escherichia coli*‐induced pneumonia and improve recovery from ventilator‐induced lung injury in rats, and this response was superior to SDC2^−^ cells and comparable to the heterogeneous MSC population *in vivo* [20]. Similarly, human umbilical cord‐derived SDC2^+^ MSCs were shown to be as effective as the total heterogeneous MSC population in reducing *E. coli*‐induced acute lung injury in rats [21]. While these studies used SDC2 as a marker for isolation of a homogeneous population of MSCs, the purpose of our study was to determine whether SDC2 plays an important role in MSC biology and function during experimental sepsis.

Syndecans are heparan sulfate transmembrane proteoglycans that interact with a large number of ligands, and these molecules play a role in many cellular signaling events related to cell adhesion, tissue repair, and inflammation [22]. Interestingly, while involved in tissue repair, the expression of SDC2 is increased in alveolar macrophages in patients with pulmonary fibrosis, and exerts antifibrotic effects in experimental models of lung fibrosis [23, 24]. In regard to inflammation, SDCs have been shown to regulate leukocyte extravasation and cytokine/chemokine function [25], and SDCs are involved in many aspects of the inflammatory response, from leukocyte recruitment to resolution of inflammation [26]. Since MSCs are also known to modulate inflammation and tissue repair, we propose that SDC2 plays an important role in MSC function during sepsis to alleviate organ dysfunction.

## Results

### SDC2 expression is higher in human MSCs compared with fibroblasts, and silencing of SDC2 alters cell growth

**Fig. 1:**
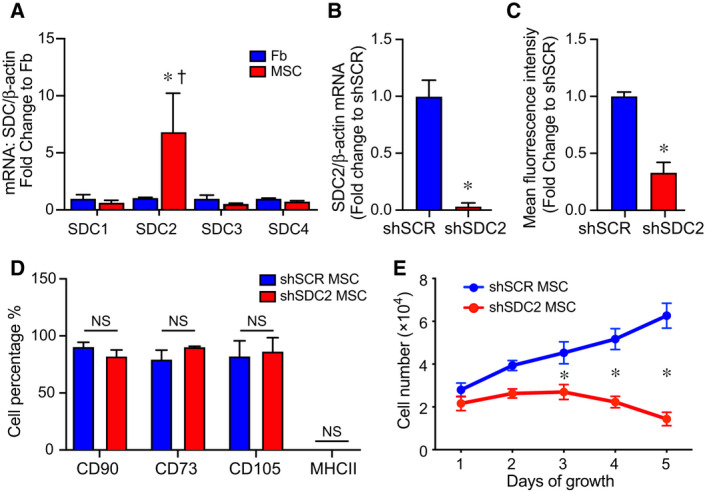
Syndecan‐2 is highly expressed in bone marrow‐derived hMSCs, and silencing results in no change in mesenchymal markers but decreased cell growth. (A) RNA was extracted from human dermal fibroblasts (Fb, blue bars) and bone marrow‐derived hMSCs (MSC, red bars), and qRT‐PCR was performed for SDC1, SDC2, SDC3, and SDC4. Data are presented as mRNA levels of SDCs normalized for β‐actin, as a fold change to Fb, mean ± SEM, *n* = 3 in each group. *P = 0.032 versus Fb, and ^†^ *P* = 0.03 SDC2 versus other SDCs. (B) RNA was extracted from scrambled control short hairpin construct (shSCR) hMSCs (blue bar) and from silencing of SDC2 (shSDC2, red bar), and qRT‐PCR was performed for SDC2. Data are presented as mRNA levels of SDC2 normalized for β‐actin, as a fold change to shSCR hMSCs, mean ± SEM, *n* = 3 in each group, *P = 0.0027 versus shSCR. (C) Flow cytometry was also performed for SDC2 in shSCR (blue bar) and shSDC2 (red bar) hMSCs. Data are presented as mean fluorescent intensity, fold change to shSCR hMSCs, mean ± SEM, *n* = 3 in each group, *P = 0.002 versus shSCR. (D) Flow cytometry was performed for CD90, CD73, CD105, and MHCII in shSCR (blue bar) and shSDC2 (red bar) hMSCs. Data are presented as percentage of cells expressing the markers in each group, mean ± SEM, *n* = 3 in each group; NS, not significant between groups. (E) shSCR hMSCs (blue dots/line) and shSDC2 (red dots/line) hMSCs were seeded on day 0, and counted daily through day 5. Data are presented as cell number × 10^4^, mean ± SEM, *n* = 3 experiments in each group, *P = 0.011 shSDC2 versus shSCR hMSC.

We analyzed the level of SDC2 in human bone marrow‐derived MSCs (hMSCs) and human dermal fibroblasts, a control mesenchymal cell. Using quantitative real‐time PCR (qRT‐PCR), the level of SDC2 mRNA in hMSCs was 6.8‐fold higher than human fibroblasts (Fig. 1A). Furthermore, when assessing the expression of SDC2 compared with other family members, SDC2 was expressed significantly higher than SDC1, SDC3, and SDC4 in bone marrow‐derived hMSCs (Fig. 1A). To explore the impact of SDC2 on hMSCs function, we silenced SDC2 using a short hairpin RNA lentiviral construct (shSDC2) compared with a scrambled control construct (shSCR). Figure 1B,C demonstrates that silencing of SDC2 resulted in decreased mRNA levels (more than 96% reduction) and protein expression (~ 67% decrease) in hMSCs. shSDC2 and shSCR hMSCs were next phenotyped using flow cytometry and exhibited comparable expression of mesenchymal markers (Fig. 1D), including CD90, CD73, and CD105. In both lines of hMSCs, they showed a very low expression of MHCII. Silencing of SDC2 in hMSCs demonstrated a significant reduction in cell growth at days 3, 4, and 5, compared with shSCR hMSCs (Fig. 1E). Furthermore, while shSCR hMSCs continued to grow over the 5 days of evaluation, the growth of shSDC2 cells was not statistically different between any of the days 1–5.

### Silencing SDC2 leads to a loss of hMSC survival benefit, failure to protect from tissue injury, and ineffective bacterial clearance in experimental sepsis

**Fig. 2:**
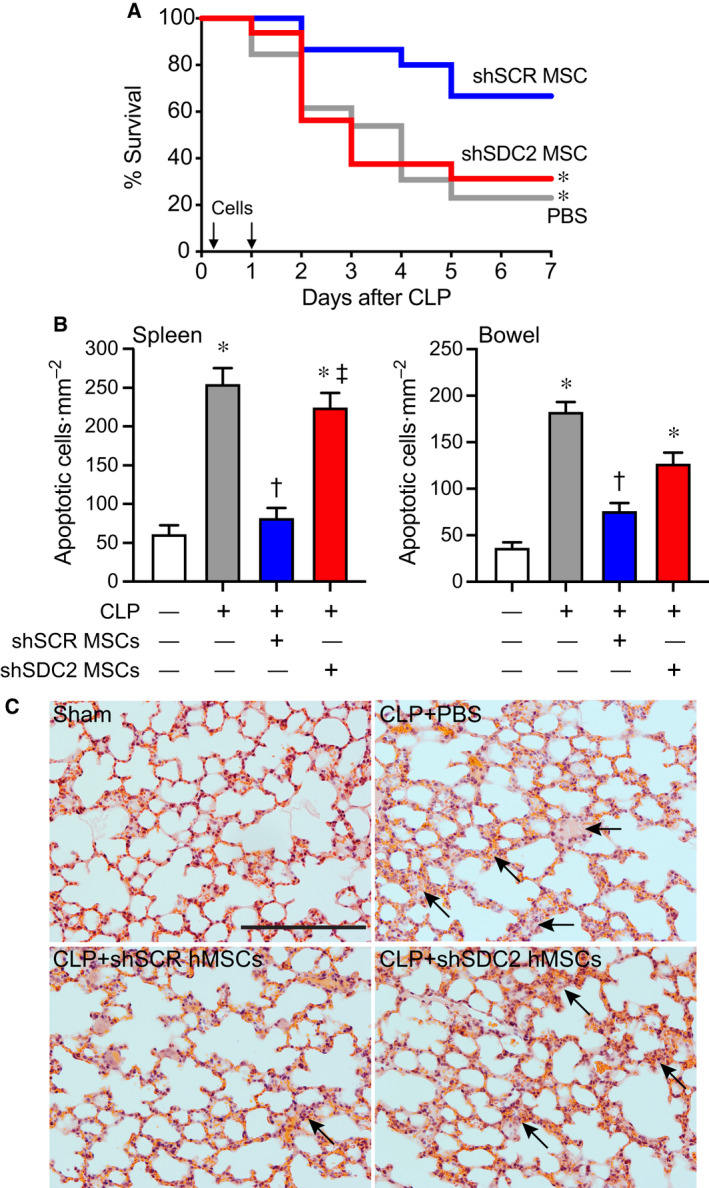
Silencing of SDC2 in hMSCs results in decreased survival and increased tissue injury and a lack of bacterial clearance when administered during sepsis. (A) Septic mice were randomly separated into three groups: PBS control (gray line, *n* = 13), shSCR hMSCs (blue line, *n* = 15), and shSDC2 hMSCs (red line, *n* = 16). All mice were subjected to CLP. 2 h after CLP, the mice received PBS (200 µL) or hMSCs (5 × 10^5^ cells in 200 µL PBS) via tail vein injection. This treatment was also repeated at 24 h after CLP. Survival of mice was monitored over 7 days, and data are presented as Kaplan–Meier survival curves, *P = 0.025 versus shSCR hMSC. (B) Tissue injury was assessed by terminal deoxynucleotide transferase‐mediated dUTP nick end‐labeling (TUNEL) staining of spleen (left panel) and bowel (right panel) tissues 48 h after sham (−) or CLP (+) surgery. Data are presented as quantification of apoptotic cells per mm^2^, mean ± SEM, from random images of fluorescent microscope (×20 objective) in sham (white bars, *n* = 10 and 13 images, respectively, from left and right panels), CLP+PBS (gray bars, *n* = 24 and 18, respectively), CLP+shSCR hMSCs (blue bars, *n* = 27 and 18 images, respectively), and CLP+shSDC2 hMSCs (red bars, *n* = 27 and 20 images, respectively). *P* < 0.0001 for spleen and bowel, with significant comparisons * versus sham, † versus PBS, ‡ versus shSCR hMSCs. (C) Lung architecture was assessed by hematoxylin and eosin staining of representative tissue sections from sham (left upper panel), CLP+PBS (right upper panel), CLP+shSCR hMSCs (left lower panel), and CLP+shSDC2 hMSCs (right lower panel). Arrows point to areas of injury. Scale bar represents 100 µm.

We next assessed the therapeutic impact of hMSC‐derived SDC2 function *in vivo*. After the induction of sepsis by cecal ligation and puncture (CLP), mice received vehicle PBS, shSCR hMSCs, or shSDC2 hMSCs. hMSCs (5 × 10^5^ cells/200 µL PBS) or vehicle (PBS 200 µL) was administered intravenously 2 h, and then again 24 h after CLP, and survival was assessed over 7 days. Mice treated with PBS alone had a survival rate of ~ 23% (Fig. 2A). Injection of shSCR hMSCs led to a marked increase in mouse survival (~ 67%), whereas the survival of mice receiving shSDC2 hMSCs was significantly diminished (~ 31%). Assessment of organ injury at 48 h after CLP or sham surgery revealed mice receiving shSDC2 hMSCs or PBS after CLP had a similar increase in apoptosis of spleen and bowel (distal small intestine), whereas mice receiving shSCR hMSCs after CLP had a blunted apoptotic response, comparable to sham surgery (Fig. 2B). Lung injury is another consequence of sepsis, and hMSCs have been shown to restore fluid clearance and have antimicrobial activity in human lungs *ex vivo* exposed to live bacterial in a pneumonia model [27]. The mice undergoing CLP showed evidence of thickening of the alveolar walls, with some alveolar collapse and edema at 48 h (Fig. 2C). While these changes were more evident in mice that received PBS or shSCD2 MSCs after CLP, overall the histological findings in the lungs were modest in this model of peritoneal sepsis.

**Fig. 3:**
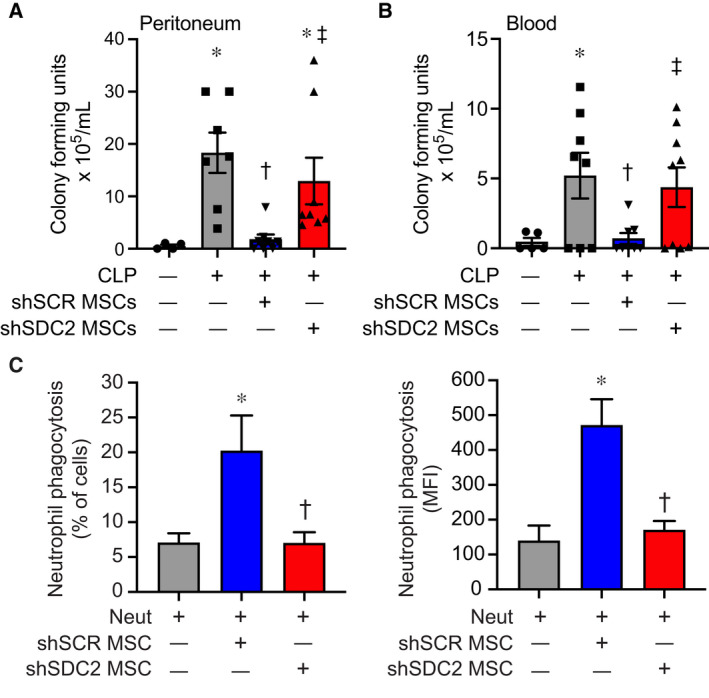
Enhanced bacterial clearance and neutrophil phagocytosis by hMSC are lost after SDC2 silencing. Assessment of bacterial colony‐forming units was assessed in the peritoneum (A) and blood (B) 48 h after sham (circles, *n* = 4 in peritoneum, *n* = 5 in blood), PBS+CLP (squares, *n* = 7 in peritoneum, *n* = 8 in blood), shSCR hMSCs+CLP (downward triangle, *n* = 8 in peritoneum and blood), and shSDC2 hMSCs+CLP (upward triangle, *n* = 8 in peritoneum, *n* = 9 in blood). Data are depicted graphically as a mean ± SEM. *P* = 0.003 (A) and *P* = 0.0004 (B) with significant comparisons * versus sham, † versus PBS, ‡ versus shSCR hMSCs. (C) Isolated neutrophils were incubated with GFP‐labeled *Escherichia* *coli* in the presence of no hMSCs (gray bars, *n* = 4 in each group), shSCR hMSCs (blue bars, *n* = 3 and 4, respectively), or shSDC2 hMSCs (red bars, *n* = 3 and 4, respectively). Data are presented as percent of neutrophils phagocytizing bacteria (left panel) or as mean fluorescent intensity (MFI) of bacteria taken up by neutrophils (right panel). Data are presented as mean ± SEM. *P* = 0.023 for % of neutrophils phagocytizing bacteria, and *P* = 0.0048 for neutrophil MFI of FITC, with significant comparisons * versus no hMSCs, and † versus shSCR hMSCs.

Bacterial clearance was also assessed at 48 h after CLP. Mice in all sepsis groups demonstrated bacteria in the peritoneum and blood. The administration of shSCR hMSCs after the onset of sepsis resulted in a significant decrease in bacteria in both the peritoneum and the blood compared with the PBS group (Fig. 3A,B). In contrast, mice receiving shSDC2 hMSCs had significantly higher numbers of bacteria in the peritoneum and blood compared with mice receiving shSCR hMSCs, analogous to mice receiving PBS alone. To further understand bacterial clearance, we assessed the influence of hMSCs on neutrophil and macrophage phagocytosis. Compared with no hMSCs, shSCR hMSCs increased the percentage of neutrophils phagocytizing bacteria (*E. coli*), and also the total amount of bacteria engulfed (Fig. 3C). In the presence of shSDC2 hMSCs, the percentage and amount of *E. coli* phagocytized by neutrophils was not different than neutrophils not exposed to hMSCs. While macrophages phagocytized *E. coli*, the effect of shSCR and shSDC2 hMSCs was not different from macrophages not exposed to hMSCs (data not shown).

### SDC2 contributes to the ability of hMSCs to modulate inflammation in sepsis

**Fig. 4:**
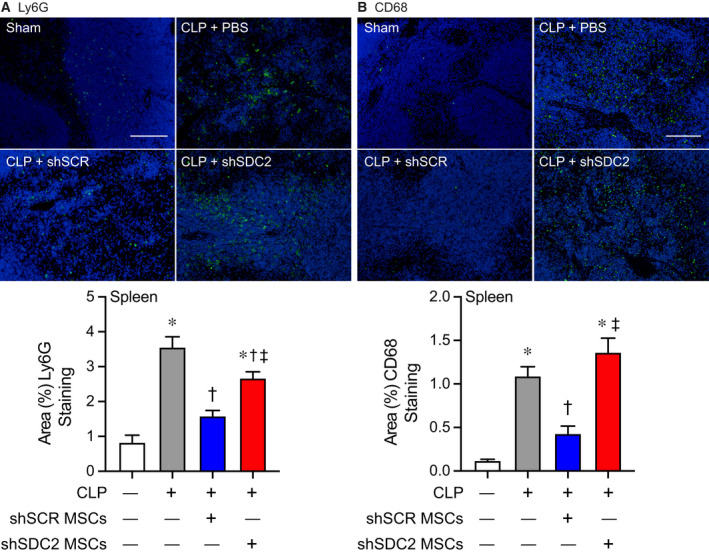
Silencing of SDC2 in hMSCs results in worse tissue Inflammation when administered during sepsis. Mice were subjected to sham or CLP surgery, and spleens were harvested at 48 h. Immunostaining (green) was performed for neutrophils (Ly6G^+^, A) and macrophages (CD68^+^, B). Representative images of the groups are provided in the upper panels. Scale bars represent 100 µm. Mice either underwent sham (white bars, images *n* = 7 and 11, respectively) or CLP surgery, and septic mice were randomly assigned to receive PBS control (gray bars, images *n* = 11 and 25, respectively), shSCR hMSCs (blue bars, images *n* = 16 and 28, respectively), or shSDC2 hMSCs (red bars, images *n* = 15 and 31, respectively). Quantitative data are presented as % area of staining for Ly6G^+^ and CD68^+^ cells, mean ± SEM. *P* < 0.0001 for Ly6G^+^ and CD68^+^, with significant comparisons * versus sham, † versus PBS, ‡ versus shSCR hMSCs.

**Fig. 5:**
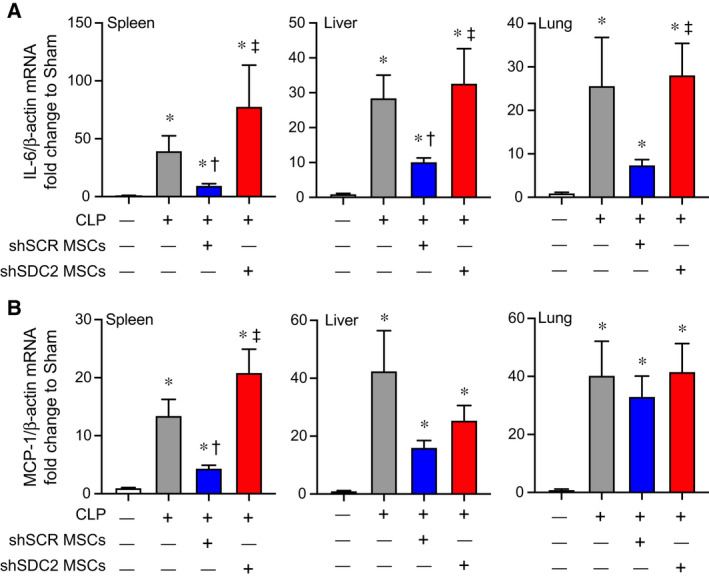
Silencing of SDC2 in hMSCs results in increased tissue expression of proinflammatory mediators. RNA was also harvested from spleen, liver, and lung tissue, and qRT‐PCR was performed for IL‐6 (A) and MCP‐1 (B). Mice undergoing CLP were randomly assigned to receive PBS control (gray bars, *n* = 20 in each group), shSCR hMSCs (blue bars, *n* = 20 in each group), or shSDC2 hMSCs (red bars, *n* = 16 for IL‐6, *n* = 18 for MCP‐1). Data are presented as mRNA levels of IL‐6 or MCP‐1 normalized for β‐actin, as a fold change to sham (white bars), mean ± SEM. *P* < 0.0001 with significant comparisons * versus sham, † versus PBS, ‡ versus shSCR hMSCs.

The spleen is an organ of functional importance during sepsis, including to help clear bacteria, and it is also susceptible to injury due to the immune response during systemic infection [28]. Thus, we assessed the infiltration of innate immune cells into splenic tissue after CLP, using immunostaining for neutrophils (Ly6G^+^) and macrophages (CD68^+^). There was increased infiltration of Ly6G^+^ neutrophils and CD68^+^ macrophages in the PBS and the shSDC2 hMSC groups after CLP, compared with the shSCR hMSC group (Fig. 4A,B). In addition, we measured the mRNA levels of the inflammatory cytokine IL‐6, which is important in the pathobiology of sepsis [29, 30], tumor necrosis factor alpha (TNF‐α), and the chemokine MCP‐1, at 24 h after CLP. These mediators are biomarkers of the inflammatory response in sepsis [31]. The level of IL‐6 mRNA was significantly higher in the shSDC2 hMSC group compared with the shSCR hMSC group, a level comparable to PBS in spleen, liver, and lung tissues (Fig. 5A). A similar pattern of MCP‐1 (Fig. 5B) and TNF‐α (data not shown) mRNA levels was seen in the spleen, with elevated levels in the shSDC2 hMSC group compared with the shSCR hMSC group. The levels of MCP‐1 and TNF‐α mRNA had a similar trend in liver and lung tissues, but the changes were not statistically significant.

**Fig. 6:**
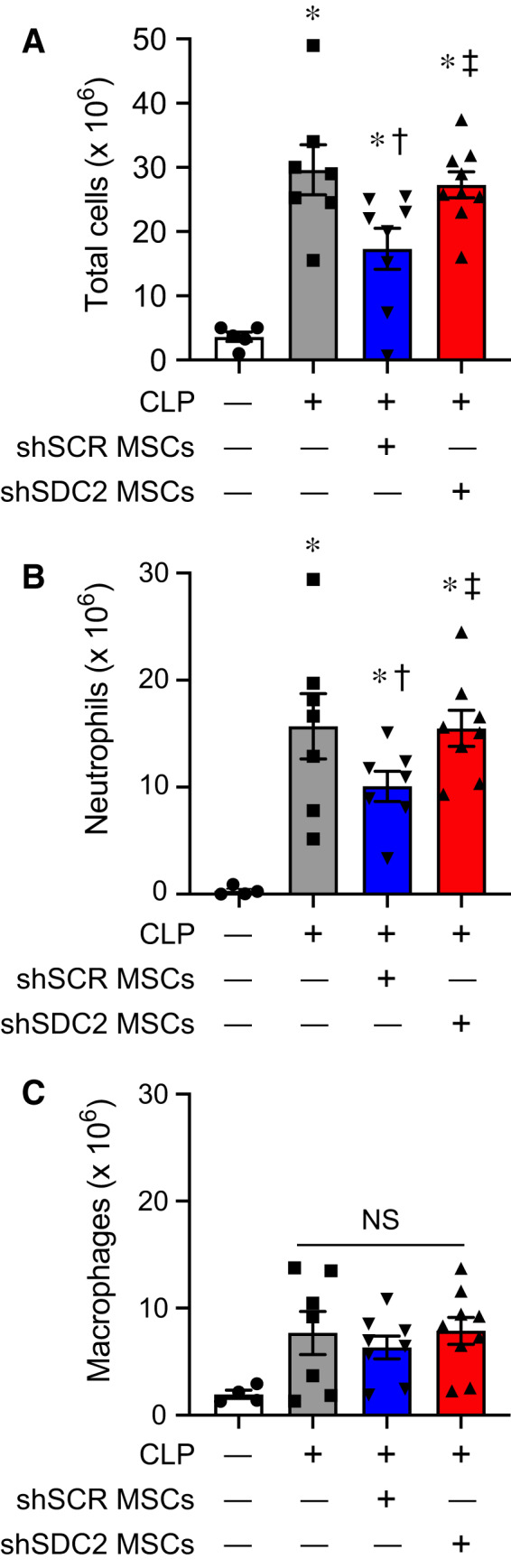
Silencing of SDC2 in hMSCs results in less efficient resolution of neutrophilic inflammation in the peritoneum when given during CLP‐induced sepsis. Total cell counts (A), innate immune neutrophils (B), and macrophages (C) were assessed in peritoneal fluid (PF) of mice undergoing sham or CLP surgery after 48 h. Mice were randomly assigned to sham (circles/white bars, *n* = 5 in A, *n* = 4 in B and C) or CLP surgery, and received PBS control (− MSCs, squares/gray bars, *n* = 7 in each group), shSCR hMSCs (+, downward triangles/blue bars, *n* = 8 in A and C, *n* = 7 in B), or shSDC2 hMSCs (+, upward triangles/red bars, *n* = 9 in A and C, *n* = 8 in B). Data are presented as cells × 10^6^, mean ± SEM. For total cells and neutrophils, *P* < 0.0001 and *P* = 0.0005, respectively, with significant comparisons * versus sham, † versus PBS, ‡ versus shSCR hMSCs. For macrophages, NS = not significant between groups.

We next assessed the inflammatory response in the peritoneum, the site of initial injury in CLP‐induced sepsis. At 48 h after CLP, the total number of cells in the peritoneal fluid was significantly decreased in the shSCR hMSC group, compared with the shSDC2 hMSC and PBS groups (Fig. 6A). Quantification of neutrophils demonstrated an analogous response, with more neutrophils in the shSDC2 hMSC group compared with the shSCR hMSC group (Fig. 6B). Finally, we assessed total macrophages (Fig. 6C) in the peritoneal fluid. In contrast to the spleen, the peritoneal fluid demonstrated no significant difference in the number of macrophages in mice receiving shSCR hMSC, shSDC2 hMSC, or PBS during CLP. We next further assessed the effect of hMSCs on macrophage subtypes and function.

### SDC2 is important for hMSCs to promote efferocytosis and macrophage polarization

**Fig. 7:**
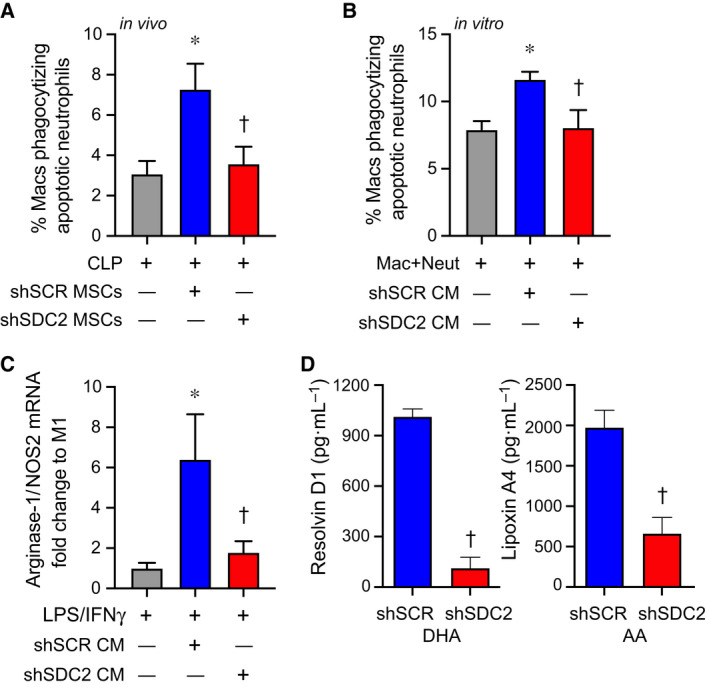
SDC2 is important for the paracrine functions of hMSCs to promote efferocytosis and macrophage polarization to an M2‐like phenotype. (A) Mice were subjected to CLP surgery and randomly assigned to receive PBS control (gray bar, *n* = 6), shSCR hMSCs (blue bar, *n* = 7), or shSDC2 hMSCs (red bar, *n* = 6). Efferocytosis was assessed in the peritoneal fluid of septic mice by flow cytometry. Data are presented as the percentage of macrophages phagocytizing apoptotic neutrophils, mean ± SEM. *P* = 0.015, with significant comparisons * versus PBS and † versus shSCR hMSC. (B) Next, we performed the efferocytosis assay *in vitro*. Macrophages were exposed to apoptotic neutrophils in the presence of PBS (gray bar, *n* = 5), CM from shSCR hMSCs (blue bar, *n* = 5), or CM form shSDC2 hMSCs (red bar, *n* = 5). Data are presented as the percentage of macrophages phagocytizing apoptotic neutrophils, mean ± SEM. *P* = 0.021, with significant comparisons * versus PBS and † versus shSCR hMSC. (C) M0 macrophages were stimulated with IFN‐γ (10 ng·mL^−1^) and LPS (10 ng·mL^−1^) to induce an M1 phenotype. At the time of LPS/IFN‐γ stimulation, the cells were exposed to PBS (gray bar, *n* = 4), shSCR CM (blue bar, *n* = 4), or shSDC2 CM (red bar, *n* = 4). Macrophage RNA was harvested, and qRT‐PCR was performed to assess arginase‐1/NOS2 ratio as a marker of macrophage polarization, with an increase consistent with M2‐like phenotype. Data are presented as mean ± SEM. *P* = 0.0048, with significant comparisons * versus PBS and † versus shSCR hMSC. (D) shSCR hMSCs (blue bars, *n* = 5) and shSDC2 hMSCs (red bars, *n* = 5) were exposed to SPM substrates DHA or AA for 24 h, and then, the CM from the cells was analyzed by ELISAs for resolvin D1 (left) or lipoxin A4 (right), respectively. Data are presented as mean ± SEM. *P* < 0.0001, with significance comparison † versus CM of shSCR hMSCs.

Macrophages and neutrophils work in concert to eliminate pathogens, and after bacterial clearance is initiated, macrophages phagocytize apoptotic neutrophils and cellular debris (efferocytosis), while concomitantly shifting from an M1‐like proinflammatory phenotype to an M2‐like proresolution phenotype [2, 3, 32, 33]. To further assess the impact of hMSC‐derived SDC2 on macrophage function, we assessed efferocytosis, an important process during the resolution of inflammation [34]. Administration of shSCR hMSCs at 2 and 24 h after CLP increased the clearance of apoptotic neutrophils by macrophages in the peritoneum at 48 h (Fig. 7A). However, mice receiving shSDC2 hMSCs had a level of efferocytosis similar to mice receiving PBS. We also explored this concept *in vitro*, utilizing the conditioned medium (CM) of hMSCs and their ability to promote macrophage phagocytosis of apoptotic neutrophils in culture. CM from shSCR hMSCs was able to increase macrophage phagocytosis of apoptotic neutrophils, while shSDC2 CM had significantly reduced efferocytosis (Fig. 7B). These data suggest that silencing of SDC2 in hMSCs promotes a loss of efferocytosis, and this process relates to its paracrine actions.

Next, we assessed whether this effect on efferocytosis by the CM of hMSCs translated into a change in macrophage subtype, from an M1‐like to an M2‐like phenotype. We took nonactivated murine macrophages (M0) and stimulated them with LPS and IFN‐γ to induce an M1‐like proinflammatory phenotype, and at the same time added CM from shSCR or shSDC2 hMSC. To identify macrophage subtypes, we used markers of macrophage arginine metabolism to characterize M1‐like macrophages (expressing more NOS2) and M2‐like macrophages (expressing more arginase‐1) [35]. A ratio of arginase‐1/NOS2 was used as a biomarker of macrophage subtype. After 48 h of stimulation with LPS and IFN‐γ, in the presence or absence of hMSC CM, macrophage RNA was harvested and qRT‐PCR performed to assess arginase‐1/NOS2 ratio. Figure 7C demonstrates that CM from shSCR hMSCs promoted an M2‐like phenotype, with an increased ratio of arginase‐1/NOS2, while in the presence of CM from shSDC2 hMSC, the arginase‐1/NOS2 ratio was comparable to M1‐like macrophages (no CM). Specialized proresolving lipid mediators (SPMs) are known to orchestrate the resolution of inflammation, involving efferocytosis and polarization to an M2‐like proresolution macrophage phenotype, and SPMs also have anti‐inflammatory properties [36, 37, 38, 39, 40]. Resolvin D1 and lipoxin A4, SPMs expressed in MSCs, have been shown to regulate the inflammatory response during models of sepsis and acute lung injury [18, 41]. Interestingly, when we assessed these SPMs in the CM of hMSCs, we found that the production of both resolvin D1 and lipoxin A4 was significantly reduced in shSDC2 MSCs compared with shSCR MSCs (Fig. 7D).

### SDC2 is important for the ability of hMSC‐derived extracellular vesicles to promote macrophage polarization and efferocytosis

**Fig. 8:**
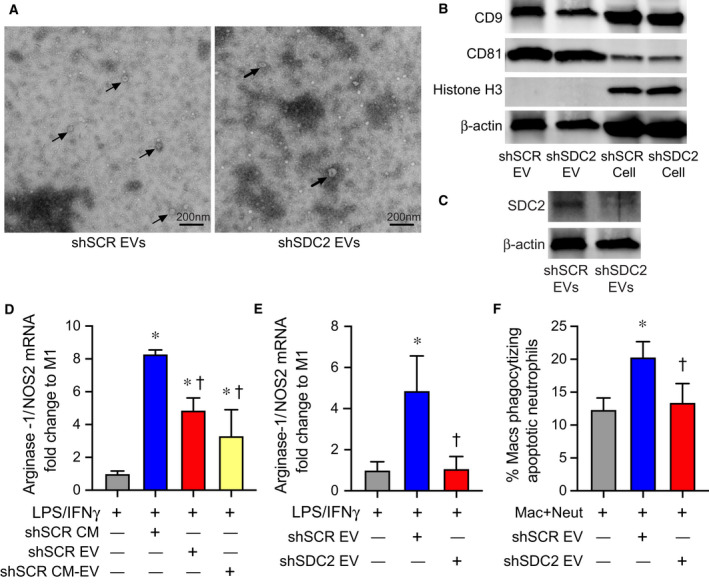
Extracellular vesicles secreted by shSDC2 hMSCs lose the ability to promote efferocytosis and macrophage polarization to an M2‐like phenotype. (A) TEM was performed on EVs harvested from shSCR hMSCs (left panel) and shSDC2 hMSCs (right panel). Arrows point to representative EVs. Scale bar represents 200 nm. (B) EVs isolated from shSCR hMSCs and shSDC2 hMSCs were characterized by western blot analyses, using antibodies to CD9 and CD81 as markers of EVs, and histone H3 as a negative control of EVs (upper panel). Total cell protein lysate was loaded as a control, and β‐actin was used to signify total protein content. (C) EVs isolated from shSCR hMSCs and shSDC2 hMSCs were also characterized by western blot analyses, using an antibody to SDC2 (lower panel). Total cell protein lysate was loaded as a control, and β‐actin was used to signify total protein content. (D) M0 macrophages were stimulated with IFN‐γ (10 ng·mL^−1^) and LPS (10 ng·mL^−1^) to induce an M1 phenotype. At the time of LPS/IFN‐γ stimulation, the cells were exposed to PBS (gray bar, *n* = 4), shSCR CM (blue bar, *n* = 4), shSCR EV (red bar, *n* = 5), or shSCR CM depleted of EVs (shSCR CM‐EV, yellow bar, *n* = 3). Macrophage RNA was harvested, and qRT‐PCR was performed to assess the arginase‐1/NOS2 ratio as a marker of macrophage polarization. Data are presented as mean ± SEM. *P* < 0.0001, with significant comparisons * versus PBS and † versus shSCR hMSC. (E) M0 macrophages were stimulated with IFN‐γ (10 ng·mL^−1^) and LPS (10 ng·mL^−1^) to induce an M1 phenotype. At the time of LPS/IFN‐γ stimulation, the cells were exposed to PBS (gray bar, *n* = 5), shSCR EV (blue bar, *n* = 4), or shSDC2 EV (red bar, *n* = 5). Macrophage RNA was harvested, and qRT‐PCR was performed to assess the arginase‐1/NOS2 ratio as a marker of macrophage polarization. Data are presented as mean ± SEM. *P* = 0.005, with significant comparisons * versus PBS and † versus shSCR hMSC. (F) Macrophages were exposed to apoptotic neutrophils in the presence of PBS (gray bar, *n* = 7), shSCR EV (blue bar, *n* = 7), or shSDC2 EV (red bar, *n* = 7). Data are presented as the percentage of macrophages phagocytizing apoptotic neutrophils, mean ± SEM. *P* = 0.0027, with significant comparisons * versus PBS and † versus shSCR hMSC.

An important component of the paracrine actions of MSCs is extracellular vesicles (EVs) [42]. Thus, we next explored the capacity of EVs derived from shSCR and shSDC2 hMSC to modulate macrophage polarization and efferocytosis to initiate the resolution of inflammation. We harvested EVs from hMSCs as described [43]. The isolation and characterization of EVs were in accordance with the 2018 Minimal Information for Studies of Extracellular Vesicles (MISEV) [44], as outlined by the International Society for Extracellular Vesicles. In the present studies, EVs represented a heterogeneous vesicle population that occupy a diameter of < 200 nm, express established EV‐associated markers including CD9 and CD81, and adhere to the typical biconcave features of EVs (Fig. 8A). Exosomes comprised a subpopulation of EVs. Beyond establishing the presence of known markers for EVs from both shSCR and shSDC2 hMSCs (Fig. 8B), we importantly demonstrated that histone H3 (not released in EVs [45]) was only evident in the cells and not the EVs. As a whole, these data confirm the isolation and purification of EVs from hMSCs. Moreover, SDC2 was present in shSCR EVs (Fig. 8C) and was reduced in shSDC2 EVs. We exposed macrophages (stimulated with IFN‐γ and LPS) to full CM, EVs, or the soluble fraction of CM (EV deficient) from shSCR hMSCs. In the experiments using EVs, a cell equivalent number of EVs from shSCR or shSDC2 hMSCs was administered, unless stated otherwise. Notably, EVs from shSCR hMSCs promoted polarization of macrophages from M1‐like to M2‐like phenotype (Fig. 8D). Although less dramatically, the soluble fraction also induced polarization to an M2‐like phenotype. We next assessed the impact of EVs from shSDC2 hMSCs, and shSCR hMSCs, on macrophage polarization and efferocytosis. EVs from shSDC2 hMSCs lost the ability to polarize M1‐like to M2‐like macrophages, in comparison with EVs from shSCR hMSCs (Fig. 8E). Moreover, EVs from shSCR hMSCs were able to increase efferocytosis of apoptotic neutrophils by macrophages (Fig. 8F), but after silencing SDC2, this function of EVs from shSDC2 hMSCs was lost.

### Silencing of SDC2 in hMSCs results in decreased EV production

**Fig. 9:**
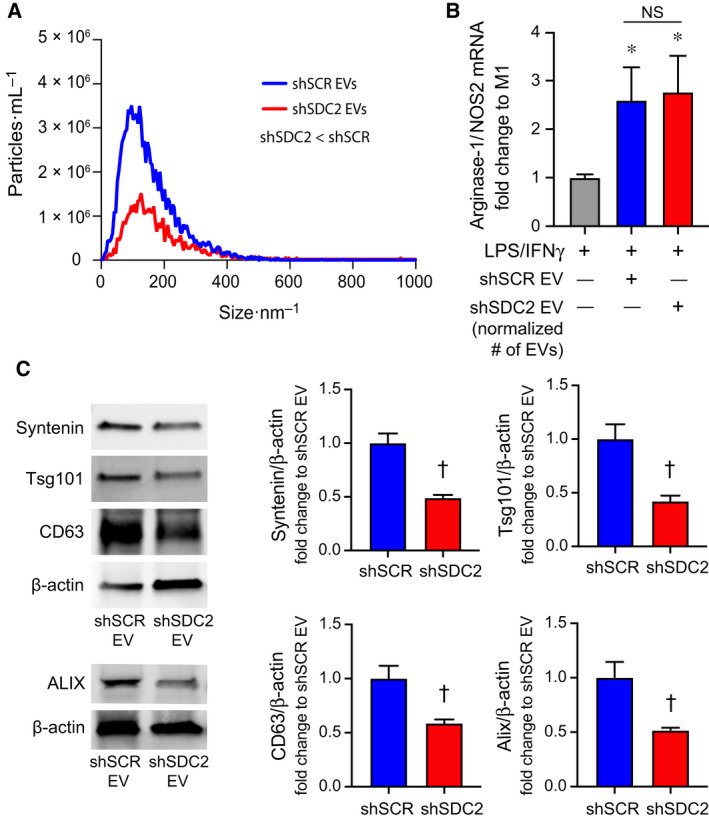
Silencing of SDC2 in hMSC reduces EV production. A) EVs were counted by NTA after harvesting EVs from an equivalent number of shSCR hMSCs (blue line, *n* = 3) and shSDC2 hMSCs (red line, *n* = 3). Data are presented as the mean of the experiments, particles per mL over the size distribution per nm, assessed by the area under the curve. *P* = 0.0005, with significant difference between shSCR and shSDC2 EVs. (B) M0 macrophages were stimulated with IFN‐γ (10 ng·mL^−1^) and LPS (10 ng·mL^−1^) to induce an M1 phenotype. At the time of LPS/IFN‐γ stimulation, the cells were exposed to PBS (gray bar, *n* = 7), shSCR EV (blue bar, *n* = 8), or shSDC2 EV (number normalized to shSCR EV, red bar, *n* = 7). Macrophage RNA was harvested, and qRT‐PCR was performed to assess the arginase‐1/NOS2 ratio as a marker of macrophage polarization. Data are presented as mean ± SEM. *P* = 0.0008, with significant comparisons * versus PBS. NS, not significant. (C) EVs isolated from shSCR hMSCs and shSDC2 hMSCs were characterized by western blot analyses (left panels), using antibodies to syntenin (*n* = 4), Tsg101 (*n* = 4), CD63 (*n* = 4), and ALIX (*n* = 3), markers of exosome biogenesis. β‐Actin was used to normalize for total protein content. The western blot data were quantified protein/β‐actin, fold change to shSCR (right panels), and presented as mean ± SEM. *P* = 0.0017, syntenin; *P* = 0.0078, Tsg101; *P* = 0.016, CD63; and *P* = 0.031, ALIX. Significant comparison † versus shSCR.

To assess the impact of SDC2 on EV production, we next performed nanoparticle tracking analysis (NTA). EV production was dramatically reduced in hMSCs silenced for SDC2 (Fig. 9A). Specifically, these data demonstrated that the EVs released from shSCR hMSCs were > 2.5‐fold greater than those from shSDC2 hMSCs. To determine whether fewer EVs are contributing to the alteration in shSDC2 paracrine function, we repeated the experiment shown in Fig. 8E, but instead of using EVs from an equivalent number of shSCR and shSDC2 hMSCs, we normalized to the number of EVs. As demonstrated in Fig. 9B, when we used an analogous number of EVs, both shSDC2 (1.5 × 10^6^ cell equivalents) and shSCR (0.5 × 10^6^ cell equivalents) hMSC EVs were able to polarize cells from an M1‐like to an M2‐like phenotype to a comparable degree. To further investigate EVs in shSDC2 hMSCs, we performed immunoblotting of EVs to assess key EV‐associated markers, using β‐actin expression for normalization. The expression of syntenin, Tsg101, CD63, and ALIX were all decreased in EVs of shSDC2 hMSCs compared with shSCR hMSCs (Fig. 9C). Taken together, these data suggest that decreased EV production, and fewer EVs in the CM of shSDC2 hMSCs, compared with shSCR hMSCs, contributed to the aberrant paracrine function.

## Discussion

Syndecan family members are transmembrane proteoglycans, with heparan sulfate extracellular domains that can be released from the cell membrane by sheddase enzymes [26, 46]. Both the expression levels and the proteolytic cleavage of extracellular domains of SDCs are known to be upregulated during inflammatory responses [26]. Circulating levels of soluble SDC1 and SDC3 have been reported to be increased during critical illnesses (including sepsis) compared with control patients [47]. By regulating leukocyte extravasation and cytokine/chemokine function, SDCs have a role in the regulation of the inflammatory response, from leukocyte recruitment to the resolution of inflammation [26]. Recently, using SDC2 as a marker on the surface of hMSCs, investigators identified a population of cells that was beneficial against bacterial pneumonia and ventilator‐induced lung injury [20, 21]. This population of SDC2^+^ cells was as effective as standard heterogeneous hMSCs to decrease severity, and improve recovery, from these acute lung injury models. Administration of SDC2^+^ hMSCs during experimental sepsis was also reported to be advantageous compared with a vehicle control [48]. Given that human MSCs expressing SDC2 on their cell surface were comparable to but not more effective than the heterogeneous MSC population, we sought to further elucidate the importance of endogenous SDC2 on hMSC function.

Syndecans in mammals are expressed in cell‐ and tissue‐specific patterns, and SDC2 is known to be expressed in mesenchymal cells [49]. Interestingly, we demonstrated that the expression of SDC2 is more than sixfold higher in bone marrow‐derived hMSCs than in control mesenchymal fibroblasts, and SDC2 is much more highly expressed than SDC1, SDC3, or SDC4 in hMSCs. Silencing of SDC2 resulted in a loss of survival benefit, more tissue cell death, and less bacterial clearance when administered after the onset of polymicrobial sepsis compared with shSCR hMSCs. While the administration of shSCR hMSCs resulted in a decrease in the infiltration of neutrophils (Ly6G^+^) and macrophages (CD68^+^) into splenic tissue during sepsis, this decrease in innate immune cell infiltration was not evident in mice receiving shSDC2 hMSCs. Evaluation of the inflammatory response in the peritoneum (site of injury) revealed a similar decrease in neutrophils in mice receiving shSCR hMSCs after the onset of sepsis, and this response was lost in mice receiving shSDC2 hMSCs.

With evidence that the inflammatory response was not resolving as efficiently after receiving shSDC2 hMSCs, compared with shSCR hMSCs, we further assessed the phagocytosis of apoptotic neutrophils by macrophages (efferocytosis), a critical process during the resolution of inflammation [34, 50]. Here, we found that efferocytosis was significantly greater in mice receiving shSCR hMSCs than mice receiving shSDC2 hMSCs. The clearance of apoptotic neutrophils by macrophages is associated with a shift from an M1‐like proinflammatory to an M2‐like proresolution phenotype [2, 3, 33]. Thus, even though the overall number of peritoneal macrophages was not different between the groups, we hypothesized a shift in macrophage phenotype. The CM from shSCR hMSCs was able to promote the conversion of M1‐like macrophages to M2‐like macrophages, and this effect was not present when using the CM of shSDC2 hMSCs. Furthermore, we demonstrate for the first time that the production of resolvin D1 and lipoxin A4, SPMs known to promote efferocytosis and polarization of macrophages to a proresolution phenotype, was decreased in the CM of shSDC2 compared with shSCR hMSCs. SPMs are dependent on lipoxygenase (LOX) enzymes for their biosynthesis [50], and exposure of human and mouse MSCs to a LOX inhibitor (baicalein), or silencing of the 5‐LOX and 12/15‐LOX enzymes in mouse MSCs resulted in a blunted effect on neutrophil phagocytosis of bacteria and a loss of survival benefit during peritoneal sepsis [18]. Thus, the defect in SPM production has important consequences on the function of shSDC2 hMSCs during sepsis.

The ability of MSCs to modulate an inflammatory response and to protect tissue from injury, or to promote tissue repair, is largely via their paracrine actions [6, 51, 52]. An important component of the paracrine actions of MSCs occurs through EVs, and MSC‐derived EVs have been shown to be beneficial in sepsis [52]. Uptake of EVs by macrophages is able to induce a switch from the M1‐like to M2‐like phenotype [53, 54], and this modulation of macrophages has been shown to be important in experimental models of infection, inflammatory organ injury, acute lung injury, and pulmonary hypertension [52, 54, 55]. Interestingly, biodistribution of EVs accumulates mainly in organs such as the spleen, liver, and lung [56], organs in which the inflammatory mediators IL‐6 and MCP‐1 were decreased after administration of shSCR hMSCs, compared with shSCD2 hMSC. It is uncertain whether there is a potential for EV homing to specific organs during disease, although the route of administration influences the biodistribution [56]. Moreover, Mansouri and colleagues also demonstrated that MSC‐derived exosomes/EVs were able to reprogram myeloid cells in the bone marrow, leading to lower proinflammatory monocytes in the lung after administration of bleomycin [57]. Thus, the effect of EVs on bone marrow‐derived myeloid cells may also provide an immunomodulatory mechanism by which EVs have a systemic response.

In the present study, the paracrine actions of shSCR hMSCs to induce the conversion of M1‐like to M2‐like macrophages were in part related to EVs in the CM, along with soluble factors. However, EVs harvested from shSDC2 hMSCs (from an equivalent number of cells as shSCR hMSCs) failed to induce a change in macrophage phenotype. Additionally, the EVs from shSDC2 hMSCs were not able to increase the phagocytosis of apoptotic neutrophils, as seen with shSCR EVs.

In an effort to understand the mechanism behind this abnormal response of EVs from shSDC2 hMSCs, we assessed the number of EVs present in the CM of an equivalent number of cells in each groups. We noted there was more than ~ 2.5‐fold fewer EVs in the CM of shSDC2 hMSCs compared with shSCR hMSCs, and when we normalized for the number of EVs, the paracrine actions of shSDC2 EVs were analogous to shSCR EVs in converting M1‐like to M2‐like macrophages. Previously, it has been shown that the cytosolic adaptor syntenin connects to ALIX via the heparan sulfate proteoglycans of SDC1 and SDC4 and then interacts with other endosomal sorting complex required for transport (ESCRT) machinery to support membrane budding and biogenesis of exosomes (subpopulation of EVs) [58, 59]. Interestingly, we found that silencing of SDC2 in hMSCs resulted in decreased expression of syntenin, ALIX, Tsg101, and CD63, established EV‐associated markers. Taken together, these data support the concept that SDC2 is critical for the production of EVs in hMSCs, and we hypothesize that decreased numbers of EVs from shSDC2 hMSCs may contribute to decreased efferocytosis, loss of macrophage polarization to the M2‐like phenotype, and altered resolution of inflammation. Since SDC1 and SDC4 are known to influence the cargo of EVs [60, 61, 62], we cannot exclude the impact of SDC2 on the composition of MSC‐derived EVs and this influence on sepsis pathobiology.

In conclusion, this study demonstrates an important role for endogenous SDC2 on bone marrow‐derived hMSC function (both cellular and paracrine actions) during experimental sepsis. Beyond the ability of hMSC to promote bacterial clearance by neutrophils, SDC2 is important to allow prompt resolution of inflammation resulting in less tissue injury and improved survival. The ability of hMSCs to enhance the clearance of apoptotic neutrophils from the peritoneum, and transition of macrophages from an M1‐like proinflammatory phenotype to an M2‐like proresolution phenotype, is lost after silencing of SDC2 in the cells. In addition, the paracrine actions of hMSC‐derived EVs contributes to efferocytosis and M2‐like polarization of macrophages *in vitro*, and these actions are related in part to the impact of SDC2 on cellular EV production. Collectively, these data advance our understanding of how SDC2 promotes hMSC function during experimental polymicrobial sepsis.

## Materials and methods

### Cells

Primary human bone marrow‐derived mesenchymal stromal cells (hMSCs) were obtained from the Institute for Regenerative Medicine, Texas A&M Health Science Center. hMSCs were cultured in MEMα (Gibco, Gaithersburg, MD, USA) supplemented with 20% FBS, and used at passages 5–6. Control mesenchymal cells were human dermal fibroblasts.

### Lentivirus silencing of SDC2 in hMSC

The vector for SDC2 (shSDC2), target sequence 5′‐GTCATTGCTGGTGGAGTTATT‐3′ (TRCN0000298635), and scrambled control (shSCR) construct (SHC016) were purchased from Sigma‐Aldrich (St. Louis, MO, USA). For production of lentiviral particles, a second‐generation packaging mix and LentiFectin™ Transfection Reagent (Applied Biological Materials, Richmond, BC, Canada) were used. shSDC2 and shSCR lentiviral particles were added to hMSCs for 24 h, followed by selection using puromycin (10 μg·mL^−1^) as described [18].

### Assessment of SDC2 silencing by qRT‐PCR and flow cytometry

Total RNA was extracted from shSDC2‐ and shSCR‐infected hMSCs, and qRT‐PCR was performed as described [63, 64] using the human primers of SDC2 forward 5′‐CAACATCTCGACCACTTCCA‐3′ and reverse 5′‐TGGGTCCATTTTCCTTTCTG‐3′. qRT‐PCR of β‐actin was used for normalization of SDC2 expression by the comparative Ct method using primers of human β‐actin forward 5′‐AGGCACCAGGGCGTGAT‐3′ and reverse 5′‐GCCCACATAGGAATCCTTCTGAC‐3′. Cells were harvested after silencing, and flow cytometry was performed, using the CD362(SDC2)‐PE antibody (Table 1). The cells were then assessed using a BD FACSCanto II, and analyzed by flowjo software (Becton Dickinson and Company, Franklin Lakes, NJ, USA).

**Table 1:** Antibodies used for flow cytometry, western blot analyses, and immunofluorescent staining.

Flow cytometry				
Target	Company	Catalog number	Clone	Fluorophore
Syndecan‐2	MACS	130‐107‐480	REA468	PE
CD90	Biolegend	328115	5E10	AF647
CD73	Biolegend	344015	AD2	FITC
CD105	Biolegend	323207	43A3	APC
HLA DR	Biolegend	307609	L243	APC
Ly6G	Biolegend	127613	1A8	APC
Ly6G	Biolegend	127606	1A8	FITC
CD11b	BD bioscience	557397	M1/70	PE
F4/80	Biolegend	123116	BM8	APC

Western blot analyses				
Target	Company	Catalog number	Isotype	Host species
Syndecan‐2	Abcam	ab191062	IgG	Rabbit
ALIX	Abcam	ab117600	IgG_1_	Mouse
Tsg101	Abcam	ab30871	IgG	Rabbit
Syntenin‐1	Santa Cruz	sc‐515538	IgG_2a_	Mouse
CD63	Santa Cruz	sc‐5275	IgG_1_	Mouse
CD81	Santa Cruz	sc‐166029	IgG_2b_	Mouse
CD9	Santa Cruz	sc‐13118	IgG_1_	Mouse
β‐actin	Santa Cruz	sc‐47778	IgG_1_	Mouse
Histone H3	Abcam	ab1791	IgG	Rabbit

Immunofluorescent staining				
Target	Company	Catalog number	Isotype	Host species
Ly6G	Biolegend	127602	IgG_2a_, κ	Rat
CD68	Abcam	ab125212	IgG	Rabbit

### Growth curve

Seventy‐two hours after puromycin selection, shSCR and shSDC2 hMSCs were plated in 35‐mm dishes at a density of 2 × 10^4^ cells/35‐mm dish (day 0). The medium was changed every other day. The cell number was counted daily, from day 1 to day 5.

### Cecal ligation and puncture

C57BL/6 male mice, 6–8 weeks of age, underwent CLP as described [17, 18, 19, 65], with two‐thirds of the cecum ligated and punctured with two 21‐gauge holes. In sham experiments, surgery was performed, without CLP. The mice received hMSCs (5 × 10^5^ cells/200 µL PBS) or vehicle (PBS 200 µL) via intravenous administration at 2 h after CLP, and then again at 24 h after CLP (5 × 10^5^ cells/200 µL PBS or PBS 200 µL only). The mice were sacrificed at 24–48 h after CLP, or they were monitored over 7 days to determine survival.

### Bacterial clearance

Peritoneal fluid and blood were drawn 48 h after CLP. Serial dilutions of whole blood and peritoneal fluid were performed and then incubated overnight at 37 °C on LB agar plates. CFUs of bacteria were counted and calculated as described [17].

### Flow cytometry and efferocytosis of peritoneal cells

Peritoneal lavage was performed 48 h after CLP or sham surgery. Cells from the recovered fluid were stained with antibodies targeting Ly6G‐APC and CD11b‐PE to identify neutrophils, and F4/80‐APC to identify macrophages [19]. For *in vivo* efferocytosis [18], peritoneal cells were stained with F4/80‐APC antibody. After washing with 1× PBS, the cells were permeabilized with Cytofix/Cytoperm (BD Biosciences, Billerica, MA, USA) and stained intracellularly with Ly6G‐FITC antibody. The cell population positive for both F4/80‐APC and Ly6G‐FITC by flow cytometry was identified as macrophages phagocytizing apoptotic neutrophils (efferocytosis). The antibodies used for flow cytometry are detailed in Table 1.

### Histology and immunohistochemistry

Mice were sacrificed 48 h following CLP or sham surgery, and organs were harvested and fixed in 10% formalin, processed, embedded in paraffin, and sectioned (5 µm). Tissue sections were assessed by hematoxylin and eosin stain, or stained for apoptotic cells using ApoAlert DNA Fragmentation Assay Kit (Clontech, Mountain View, CA, USA). Tissues were also immunostained with Ly6G and CD68 antibodies (Table 1) for assessment of neutrophil and macrophage infiltration. The area of positively stained cells was calculated per 20× objective using imagej software (National Institutes of Health, Bethesda, MD, USA) or Adobe Photoshop (Adobe Systems Incorporated, San Jose, CA, USA), respectively, and numerous random fields were assessed per tissue section.

### Assessment of organ inflammatory mediators by qRT‐PCR

The liver, spleen, and lung were harvested 24 h after CLP or sham surgery. Total RNA was extracted and qRT‐PCR performed [63, 64]. The primers used to assess inflammation were mouse IL‐6 forward 5′‐ACAAGTCGGAGGCTTAATTACACA T‐3′ and reverse 5′‐TTGCCATTGCACAACTCTTTT C‐3′, and mouse MCP‐1 forward 5′‐ACTGAAGCCAGCTCTCTCTTCCTC‐3′ and reverse 5′‐TTCCTTGGGGTCAGCACAGAC‐3′. Mouse β‐actin was used to normalize gene expression, using primers for mouse β‐actin forward 5′‐ACCAACTGGGACGATATGGAGAAGA‐3′ and reverse 5′‐TACGACCAGAGGCATACAGGGACAA‐3′.

### Preparation of hMSC conditioned medium

Human mesenchymal stromal cells were cultured to 80–90% confluence, washed with PBS, and replenished with supplement‐free MEMα. After 24 h, the CM was collected, and centrifuged at 805 ***g*** for 5 min to remove cell debris. The CM was then concentrated using an Amicon Ultra‐4 centrifugal filter units with a 3‐kDa cutoff (Millipore, Billerica, MA, USA). Aliquots of the concentrated hMSC CM were then kept at −80 °C until they were used.

### Isolation of hMSC extracellular vesicles

Growth medium of hMSCs (MEMα plus 20% FBS) was subjected to ultracentrifugation (100 000 ***g*** for 2 h at 4 °C) to deplete EVs. The EV‐depleted medium was then added to hMSCs for 36 h and collected, and HEPES solution (1 m Sigma, pH 7.4) was added at 1 : 40 dilution for a final concentration of 25 mm. The supernatant was then spun at 300 ***g*** for 10 min at 4 °C. The supernatant was again collected, filtered (0.22 µm), and spun at 2000 ***g*** for 10 min at 4 °C. Next, the supernatant was subjected to ultracentrifugation at 100 000 ***g*** for 90 min at 4 °C, and the pellet was collected. Finally, this pellet was resuspended in cold PBS and subjected to a final ultracentrifugation step (100 000 ***g*** for 90 min at 4 °C) to isolate EVs for use in the experiments [43].

### Transmission electron microscopy

Extracellular vesicles were assessed morphologically by transmission electron microscopy (TEM). An aliquot of EVs was absorbed to a formvar/carbon grid, stained with 2% uranyl acetate, and visualized on a JEOL 1200EX TEM as previously described [66].

### Nanoparticle tracking analysis

Extracellular vesicles from 1 × 10^6^ hMSCs were harvested as previously described, and then diluted in 100 µL of PBS. The size and concentration of the EVs were then determined using NTA (NanoSight LM10 System; Malvern Instruments, Westborough, MA, USA) as described previously [66, 67].

### Western blotting of EVs

Extracellular vesicles from shSCR and shSDC2 hMSCs were lysed in 1× RIPA buffer (Cell Signaling, Danvers, MA, USA) and 1× mini protease inhibitor cocktail (cOmplete™). After adding Laemmli's SDS Sample Buffer (6×; Boston BioProducts, Ashland, MA, USA), the lysed EVs were boiled at 100 °C for 5 min, and then, equal protein concentration was electrophoresed on 4–20% Mini Protein TGX Gels (Bio‐Rad Laboratories, Hercules, CA, USA). Antibodies for blotting (SDC2, ALIX, Tsg101, syntenin‐1, Histone H3, CD63, CD81, CD9, and β‐actin) are detailed in Table 1. Protein expression was assessed using imagej software.

### Isolation of murine macrophages and neutrophils

Mice were given an intraperitoneal injection of Bio‐Gel P100 polyacrylamide beads (2% solution; Bio‐Rad Laboratories) [17]. For harvesting neutrophils, after 16–17 h, the mice were anesthetized and 10 mL of sterile PBS was used to lavage the peritoneal cavity, and cells were washed and filtered through a 40‐µm nylon mesh. Macrophages were harvested 5 days after injection of the Bio‐Gel P100 polyacrylamide beads in a similar manner [18].

### Macrophage polarization assay

Murine macrophages were seeded at 2 × 10^6^ cells/60‐mm dish in Roswell Park Memorial Institute (RPMI) 1640 medium with 10% FBS, and after 2 h, the attached cells were M0 macrophages. Interferon (IFN)‐γ 10 ng·mL^−1^ and *E. coli* LPS 10 ng·mL^−1^ were added to each dish to induce M1 macrophage polarization. CM or EV equivalents from 5 × 10^5^ hMSCs, PBS, or recombinant human syndecan‐2 (R&D System, Minneapolis, MN, USA—500 ng·mL^−1^ [24]) were added to each dish. The cells were cultured for 48 h, and RNA was then extracted from the macrophages, and the expression of arginase‐1 and nitric oxide synthase (NOS)2 was assessed by qRT‐PCR. The primers used for mouse arginase‐1 were forward 5′‐ATGGAAGAGACCTTCAGCTAC‐3′ and reverse 5′‐GCTGTCTTCCCAAGAGTTGGG‐3′. The primers used for mouse NOS2 were forward 5′‐GCCACCAACAATGGCAACA‐3′ and reverse 5′‐CGTACCGGATGAGCTGTGAATT‐3′.

### Efferocytosis assay *in vitro*

Murine macrophages were harvested as described [18] and seeded at 2 × 10^6^ cells per 60‐mm dish for 2 h. The medium and unattached cells were removed, and CM or EVs from 5 × 10^5^ hMSC equivalents were added to the macrophages for another 2 h. Finally, 4 × 10^6^ apoptotic neutrophils (induced by overnight culture) were added to each dish, incubated for 1 h, and then harvested for flow cytometry. F4/80 and Ly6G antibodies were used to label macrophages and neutrophils as described [18].

### ELISA for human resolvin D1 and lipoxin A4

shSCR and shSDC2 hMSCs were plated on a 24‐well dish (50 000 cells per well). The cells were placed in Hanks' Balanced Salt Solution supplemented with 0.1% FBS, plus substrate docosahexaenoic acid (DHA, 10 µm) or arachidonic acid (AA, 10 µm). After 24 h, human resolvin D1 and lipoxin A4 were assessed by ELISA kits from Cayman Chemicals (Ann Arbor, MI, USA) as suggested by the manufacturer.

### Animals

Studies using mice were carried out in accordance with the Public Health Service Policy on the Humane Care and Use of Laboratory Animals, and approved by the Institutional Animal Care and Use Committee (IACUC) of Brigham and Women's Hospital.

### Statistical analysis

For comparisons between two groups, we used Student's unpaired *t*‐test. For EV particles per mL, the area under the curve was assessed by Student's unpaired *t*‐test. For analysis of more than two groups, one‐way or two‐way analysis of variance was performed. When data were not normally distributed, nonparametric analyses were performed using the Kruskal–Wallis test. Comparisons of mortality were made by analyzing the Kaplan–Meier survival curves, and then, log‐rank test was used to assess for differences in survival. Statistical significance was accepted at *P* < 0.05.

## Conflict of interest

The authors declare no conflict of interest.

## Author contributions

JH conceived and designed the study, contributed to collection and assembly of data, analyzed and interpreted the data, and wrote and approved the manuscript. YS approved the manuscript and gave financial support. GW and XL conceived and designed the study, analyzed and interpreted the data, and approved the manuscript. JI contributed to collection and assembly of data, data analysis and interpretation, and approval of manuscript. M‐YK, GL, SG, JN, and NH contributed to collection and assembly of data, and approval of manuscript. EA and KT analyzed and interpreted the data, and approved the manuscript. SE‐C, SK, SAM, and IOR conceived and designed the study, and approved the manuscript. MAP conceived and designed the study, contributed to collection and assembly of data, analyzed and interpreted the data, wrote the manuscript, and gave final approval and financial support.

## Peer Review

The peer review history for this article is available at https://publons.com/publon/10.1111/febs.16154.
